# Diffusion Tensor Imaging Studies of Cervical Spondylotic Myelopathy: A Systemic Review and Meta-Analysis

**DOI:** 10.1371/journal.pone.0117707

**Published:** 2015-02-11

**Authors:** Xiaofei Guan, Guoxin Fan, Xinbo Wu, Guangfei Gu, Xin Gu, Hailong Zhang, Shisheng He

**Affiliations:** Department of Orthopedics, Shanghai Tenth People’s Hospital, Tongji University School of Medicine, Shanghai, China; Toronto Western Hospital, CANADA

## Abstract

A meta-analysis was conducted to assess alterations in measures of diffusion tensor imaging (DTI) in the patients of cervical spondylotic myelopathy (CSM), exploring the potential role of DTI as a diagnosis biomarker. A systematic search of all related studies written in English was conducted using PubMed, Web of Science, EMBASE, CINAHL, and Cochrane comparing CSM patients with healthy controls. Key details for each study regarding participants, imaging techniques, and results were extracted. DTI measurements, such as fractional anisotropy (FA), apparent diffusion coefficient (ADC), and mean diffusivity (MD) were pooled to calculate the effect size (ES) by fixed or random effects meta-analysis. 14 studies involving 479 CSM patients and 278 controls were identified. Meta-analysis of the most compressed levels (MCL) of CSM patients demonstrated that FA was significantly reduced (ES -1.52, 95% CI -1.87 to -1.16, P < 0.001) and ADC was significantly increased (ES 1.09, 95% CI 0.89 to 1.28, P < 0.001). In addition, a notable ES was found for lowered FA at C2-C3 for CSM vs. controls (ES -0.83, 95% CI -1.09 to -0.570, P < 0.001). Meta-regression analysis revealed that male ratio of CSM patients had a significant effect on reduction of FA at MCL (P = 0.03). The meta-analysis of DTI studies of CSM patients clearly demonstrated a significant FA reduction and ADC increase compared with healthy subjects. This result supports the use of DTI parameters in differentiating CSM patients from health subjects. Future researches are required to investigate the diagnosis performance of DTI in cervical spondylotic myelopathy.

## Introduction

Cervical spondylotic myelopathy (CSM) is a common disease caused by chronic compression of the spinal cord secondary to spondylosis or disc degeneration [1]. It is the most common form of spinal cord dysfunction and the leading cause of spinal cord injury in individuals older than 55 years [2]. Its diagnosis is based primarily on clinical manifestations and imaging evidences. The compressed part of the spinal cord shows a specific high signal intensity (HSI) on T2-weighted MR image [3]. T2-weighted imaging alone, however, has low sensitivity for detecting the subtle structural damage of the cord in myelopathy, especially in patients with chronic onset of symptoms [4–6]. Therefore, it is difficult to evaluate the condition of the compressed spinal cord with such imaging modalities.

Diffusion tensor imaging (DTI) has been widely utilized to assess nerve microstructure by tracing water molecular diffusion in the brain [7–9]. The most commonly used invariants in DTI are fractional anisotropy (FA) and apparent diffusion coefficient (ADC). FA is an anisotropic parameter wherein 0 to 1, and values closer to 1 represent a more anisotropic structure. ADC represents water diffusion magnitude, with high ADC indicating high water mobility and few boundaries to water motion. Mean diffusivity (MD) is also used to represent the degree of diffusion motion of water molecules (regardless of direction) [10]. Although DTI is not in routine clinical use, it has been proven to be a non-invasive tool for detecting subtle damage to spinal cord that appears normal on conventional T2-weighted MR images [11]. Several recently published studies have showed that FA and ADC values changed significantly at compressive myelopathy levels compared with uncompressed levels or normal volunteers [11–13]. Besides, FA is believed to correlate with myelopathy severity and predict the postoperative neurologic improvement of CSM patients [14]. However, as clinical evidences accumulated, controversial results regarding to the DTI changes between CSM patients and healthy subjects were observed, and its diagnostic ability still remained elucidation. To determine whether DTI metrics were able to serve as candidate biomarkers of CSM, we conducted a systemic review and meta-analysis of current data to estimate the effective sizes of FA and ADC in CSM patients.

## Materials and Methods

### Data sources

DTI studies of CSM patients comparing with healthy control subjects were searched in three computerized database of Pubmed, Web of Scinece, EMBASE, CINAHL and Cochrane. The search was in accordance to the "preferred reporting items for systemic reviewers and meta-analysis" (PRISMA) statement [15]. The search terms used in the systemic screening were "cervical spondylotic myelopathy", "cervical pain", "cervical spinal cord", which were combined with the terms "diffusion tensor imaging", "fractional anisotropy" and "apparent diffusion coefficient". Articles were limited to those published up to December 2014. The reference lists of articles retrieved for inclusion in the review were hand searched to identify other relevant articles. Two reviewers performed independent screening of the titles and abstracts of the studies to identify the relevant studies.

### Selection criteria

We imposed the following criteria for inclusion into the meta-analysis: (1) peer reviewed DTI studies in English on humans comparing patients with CSM and healthy controls; (2) reported sufficient DTI measures (FA, ADC or MD) of region of interest (ROI) for effect size calculation. If studies did not report sufficient data, we emailed the corresponding author to obtain further information. The study was excluded from our analysis if the author did not respond.

### Data extraction

To perform the meta-analysis, a standardized mean difference (SMD) was defined as the effect size (ES) statistic, which was the difference between the mean of the experiment group and that of the control group. In the current study, the mean and standard deviation measures of the FA, ADC or MD were extracted from the most compressed level (MCL)/C2-C3 level in healthy controls and CSM patients. For the studies that selected multiple ROIs from different cervical levels, the weighted average effect size was calculated and integrated in the analysis. Meta-analysis of observational studies in epidemiology guidelines for conducting and reporting meta-analysis of observational studies were followed [16]. Apart from DTI measures, the following demographic, clinical and methodological variants were also extracted from the studies if available: the number of CSM and control participants, mean age, male ratio, clinical assessment, surgical treatment, scanner make, scanner field strength, numbers of diffusion gradient directions, voxel size, field of voxel, b factor and DTI post-processing software.

### Assessment of study quality

The quality of the included studies was assessed using the Newcastle-Ottawa scale[17]. The scale consists of nine items that cover three dimensions: selection (4 points), comparability (2 points), and exposure (3 points). A high score out of a total of 9 points indicates high quality.

### Statistical Method

We used STATA version 12.0 (STATA, College Station, TX, USA) meta package (version 1.86) for continuous data meta-analysis. I^2^-values were computed as a measure of in-between study heterogeneity. Thresholds for the interpretation of I^2^ were based on previous studies suggesting that 0% to 50% represented mild heterogeneity, 50% to 75% moderate heterogeneity, and 75% to 100% considerable heterogeneity [9]. We estimated the pooled SMD with a 95% confidence interval (CI) on the basis of both the fixed and random effects models. When low heterogeneity (I^2^<50%) was observed in the analysis, the pooled SMD was reported on the basis of the fixed effects models. Otherwise, random effects models were used. Publication bias was examined by visual inspection of funnel plot asymmetry and Egger test [18].

To investigate the potential modifiers of the DTI differences between CSM patients and healthy controls, meta-regression analyses were performed to exam the relationship between male ratio, mean age, scanner field strength and SMD for the FA and ADC values. The regression analyses were conducted using STATA software.

## Results

### Selected articles

**Fig 1:**
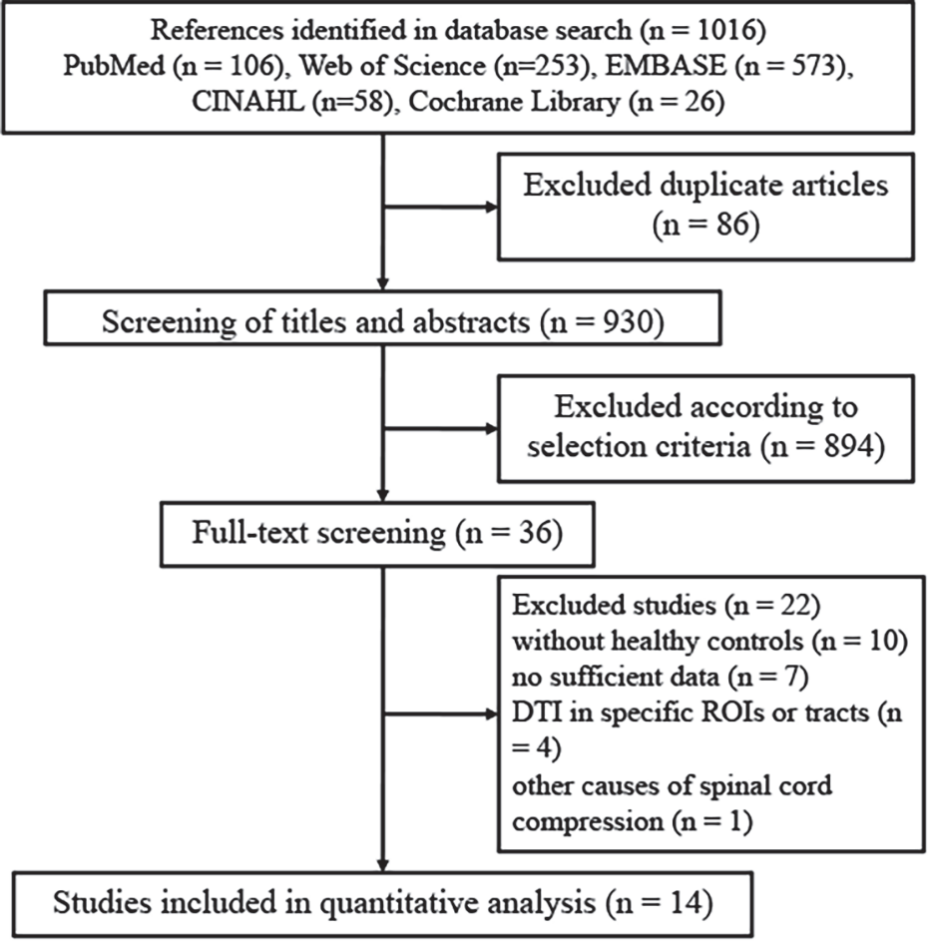
Flow of identification of relevant studies.

The method used to search relevant studies was presented in the flow diagram of Fig. 1. The initial literature search yielded 1016 original articles. The search strategy was extremely liberal, in order to capture all possible articles for inclusion in this review. After discarding 86 duplicate studies, we screened 930 studies for eligibility. From the remaining 930 potential candidates, 894 were excluded according to selection criteria. 10 studies were excluded because they did not compare CSM patients with healthy controls. 7 studies were discarded because they did not provide sufficient data to calculate the effect size. 4 studies comparing the DTI changes in specific ROIs or tracts instead of the whole spinal cord were excluded. 1 study investigating other causes of chronic spinal cord compression was excluded. Finally, 10 studies were included in our meta-analysis, involving 479 CSM patients and 278 healthy controls [12,19–31]. Demographic details of the included studies were listed in Table 1, and relevant technical factors were presented in Table 2. Assessment of study quality was summarized in Table 3.

**Table 1:** Demographic and clinical characteristics of DTI studies of CSM in meta-analysis.

Study	Year	Design	Level of evidence	Number (female)		Mean age, range (year)		Clinical assessment	Surgical treatment
				CSM	HC	CSM	HC		
Mamata	2005	Case-control	3	7(3)	11(6)	NA,26–82	37.7,30–48	NA	
Facon	2005	Case-control	3	7(3)	11(3)	48.2,30–76	36.7,NA	NA	
Xiangshui	2010	Case-control	3	84(36)	21(9)	45,16–63	43,18–60	NA	
Kim	2010	Case-control	3	8(2)	14(NA)	59.5,48–78	34,NA	NA	
Kang	2011	Case-control	3	11(5)	10(6)	67.2, NA	33.4, NA	NA	
Lee	2011	Case-control	3	21(7)	26(7)	49.6,22–67	49.6,22–67	mJOA	✓
Budzik	2011	Case-control	3	20(10)	15(7)	57.3,34–78	54.8,35–73	Self questionnaire	
Song	2011	Case-control	3	53(25)	20(6)	56,47–71	55,46–67	NA	
Uda	2013	Case-control	3	26(11)	30(15)	59.4,41–82	44.2,20–72	NA	
Wen	2013	Case-control	3	7(4)	15(NA)	56,45–67	42,36–48	mJOA	
Wen	2014	Case-control	3	45(19)	20(10)	64,43–86	52,41–62	mJOA	✓
Banaszek	2014	Case-control	3	132(78)	25(14)	53.58,18–76	45.78,27–80	NA	
Cui	2014	Case-control	3	23（8）	20(NA)	59,NA	46,NA	mJOA	
Rajasekaran	2014	Case-control	3	35(32)	40(20)	48,NA	38,NA	Nurick	

CSM, cervical spondylotic myelopathy; HC, healthy control; mJOA, modified Japanese Orthopedic Association score; NA, not available

**Table 2:** Technical details of DTI studies on ALS in meta-analysis.

Study	Year	DTI measures			Scanner make	Field-str.	DTI dir.	Voxel size(mm)	DTI proc.	ROI placement	FOV (mm)	b (mm^2^/s)
		FA	ADC	MD								
Mamata	2005	✓	✓		General Electric	1.5 T	NA	NA	NA	Sagittal	220*110	1000
Facon	2005	✓	✓		NA	1.5 T	6	1.4*1.4	DPTools	Sagittal	179*179	500
Xiangshui	2010	✓	✓		General Electric	3.0 T	15	NA	GE Functool	Axial	270*270	1000
Kim	2010				Siemens	3.0 T	12	1.5*1.5	home-made software	Sagittal	160*40	500
Kang	2011	✓		✓	Siemens	1.5 T	NA	NA	Syngo software	Axial	140*140	1000
Lee	2011	✓	✓		Philips Achieva	3.0 T	15	1.95*1.95	NA	Axial	250*224	600
Budzik	2011	✓		✓	Philips Achieva	1.5 T	25	1.56*1.56	NA	Sagittal	200*200	900
Song	2011	✓	✓		Philips Gyroscan	1.5 T	6	NA	NA	Axial	230*230	400
Uda	2013	✓		✓	Philips Achieva	3.0 T	15	1.5*1.5	NA	Axial	240*240	1000
Wen	2013	✓			Philips Achieva	3.0 T	15	0.63*0.64	Diffusion Toolkit	Axial	80*80	600
Wen	2014	✓			Philips Achieva	3.0 T	15	1.0*1.26	DTI Studio software	Axial	80*80	600
Banaszek	2014	✓	✓		General Electric	1.5 T	15	1.6*1.6	GE Functool	Axial	160*160	1000
Cui	2014	✓		✓	Philips Achieva	3.0 T	15	0.63*0.63	Diffusion Toolkit	Axial	80*36	600
Rajasekaran	2014	✓	✓		Siemens	1.5 T	12	0.86*0.86	NA	Axial	220*220	500

DTI, diffusion tensor imaging; FA, fractional anisotropy; ADC, apparent diffusion coefficient; MD, mean diffusivity; ROI, region of interest; T, Tesla; FOV, field of voxel; NA, not available

**Table 3:** Quality assessment of studies according to Newcastle-Ottawa Scale.

Study	Year	Selection				Comparability		Exposure			Total
		S1	S2	S3	S4	C1	C2	E1	E2	E3	
Mamata	2005	*	*	*	*	*	*	*	*	*	9
Facon	2005	*	*	*	*	-	-	*	*	*	7
Xiangshui	2010	*	*	*	*	*	-	*	*	*	8
Kim	2010	*	*	*	*	-	-	*	*	*	7
Kang	2011	*	*	*	*	*	-	*	*	*	8
Lee	2011	*	*	*	*	*	*	*	*	*	9
Budzik	2011	*	*	*	*	*	*	*	*	*	9
Song	2011	*	*	*	*	*	*	*	*	*	9
Uda	2013	*	*	*	*	*	-	*	*	*	8
Wen	2013	*	*	*	*	*	-	*	*	*	8
Wen	2014	*	*	*	*	*	-	*	*	*	8
Banaszek	2014	*	*	*	*	*	-	*	*	*	8
Cui	2014	*	*	*	*	*	-	*	*	*	8
Rajasekaran	2014	*	*	*	*	-	-	*	*	*	7

S1: Selection1-is the case definition adequate; S2: Selection2-representativeness of the cases; S3: Selection3-selection of controls; S4: Selection4-definition of controls. C1: Comparability1-comparability of controls for most important factor; C2: Comparability2-comparability of controls for other factors. E1: Exposure1-ascertainment of exposure; E2: Exposure2-same method of ascertainment for cases and controls; E3: Exposure3-non-response rate.

### Meta-analysis of CSM induced DTI changes at MCL

**Fig 2:**
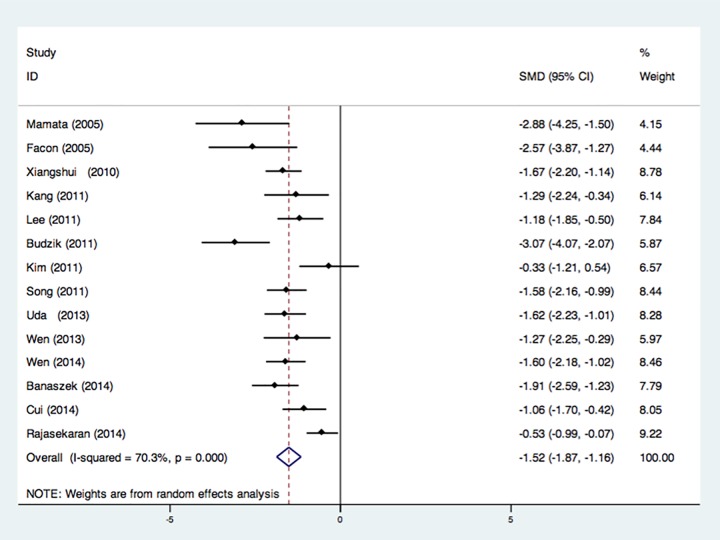
Forest plot of Standardized mean differences (SMD) for FA at most compressed level between CSM patients and healthy controls. FA was significantly reduced in CSM patients. CI indicates confidence interval.

**Fig 3:**
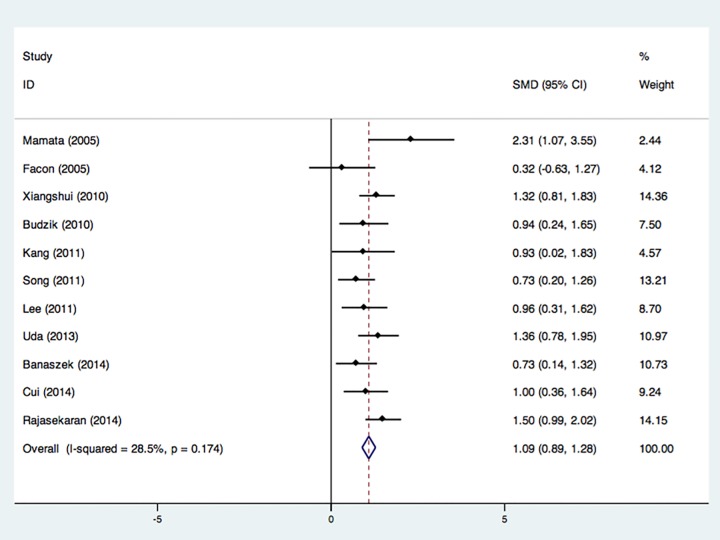
Forest plot of Standardized mean differences (SMD) for ADC at most compressed level between CSM patients and healthy controls. ADC was significantly increased in CSM patients. CI indicates confidence interval.

14 studies that recruited 380 CSM patients and 266 controls were integrated in the meta-analytical differences in FA value at MCL, and showed a significant FA decrease in CSM patients with an estimated weighted pooled disease effect size of -1.52 (P < 0.001) (Fig. 2) [12,19–31]. However, review of the heterogeneity measures revealed a moderate level of in-between study variation (I^2^ = 70.3%). 11 studies with 320 CSM patients and 215 health subjects demonstrated a significant ADC (MD) increase at the most compressed level of CSM patients (ES = 1.09, P < 0.001) with acceptable heterogeneity (I^2^ = 28.5%) (Fig. 3) [12,19,20,24–31].

### Meta-analysis of CSM induced DTI changes at C2-C3 level

**Fig 4:**
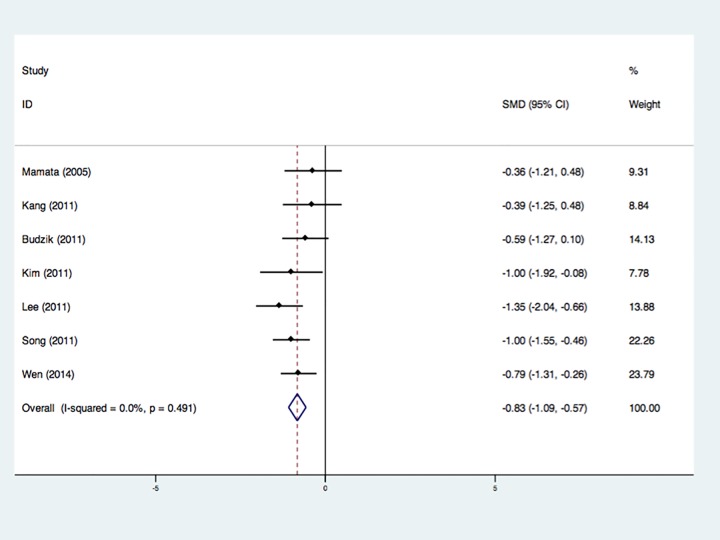
Forest plot of Standardized mean differences (SMD) for FA at C2-C3 level between CSM patients and healthy controls. FA was significantly reduced in CSM patients. CI indicates confidence interval.

7 studies involving 165 CSM patients and 112 healthy subjects were integrated using a random effect model, and revealed a decrease of FA values at C2-C3 level of CSM patients without heterogeneity (Fig. 4) [12,19,21,23,24,26,28]. 4 datasets including 77 CSM patients and 76 controls revealed no significant ADC (MD) changes at C2-C3 level between CSM patients and controls [12,24–26].

### Publication bias

For the FA reduction and ADC increase at MCL of CSM patients, the Egger test showed no evidence of publication bias (FA, P = 0.077; ADC, P = 0.944). For the decreased FA at C2-C3 level of CSM patients, the Egger test also showed no publication bias (P = 0.469).

### Meta-regression

**Fig 5:**
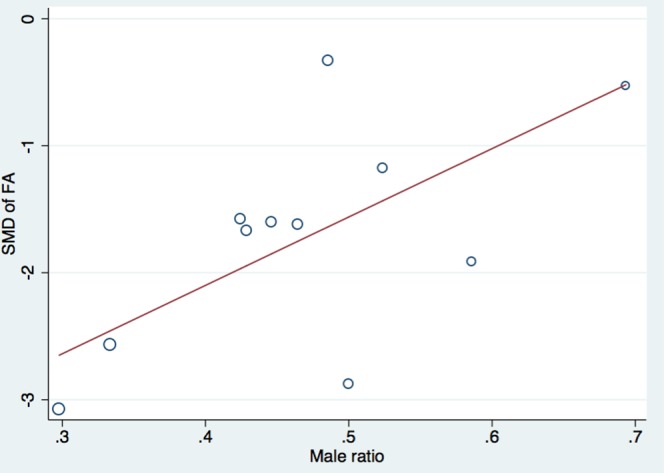
The relationship between effect size for FA and male ratio of CSM patients.

To investigate the possible modifiers of FA reduction and ADC increase in CSM patients, we conducted a meta-regression analysis with 3 modifiers: male ratio, mean age of the participants and magnetic field strength. Meta-regression analysis revealed no significant effect of mean age (P = 0.28) and magnetic field strengths (P = 0.47) on reduction of FA or increase of ADC in CSM patients. However, male ratio of CSM patients had a significant effect on reduction of FA at MCL (P = 0.03) (Fig. 5). Additionally, after reviewing all the included studies, we found a variation of ROI placement between studies. When DTI parameters were measured, the ROI were usually set to encompass the whole spinal cord on the axial plane, while four studies obtain DT images from sagittal section [12,20,23,24]. Moreover, the strongest variation of effect size in the meta-analysis was found in these studies with sagittal ROI placement. After excluding these four studies, the initial high heterogeneity was reduced in the analysis of FA values at MCL.

## Discussion

To the best of our knowledge, this is the first meta-analysis of DTI studies between CSM patients and healthy subjects. The present meta-analysis mainly showed that CSM patients had significant FA reduction and ADC increase at both the most compressed part of cervical spinal cord and C2-C3 level. In addition, meta-regression analysis was conducted to exam the potential effects of male ratio, mean age of participants, and MRI parameters on these DTI changes. Male ratio of the CSM patients and ROI placement appeared to be potential modifiers of DTI changes in CSM.

The significantly decreased FA and increased ADC demonstrated by our meta-analysis suggest the potential diagnostic ability of DTI in CSM patients. Our findings have confirmed the assumption that previous studies were unable to demonstrate significant differences because of the small number of participants. Various microstructural conditions of the compressed cervical cord, including gliosis, microcystic degeneration, demyelination and extracellular edema, may lead to increased water mobility (ADC) and decreased anisotropy (FA) [32,33]. However, previous studies suggested that the early phases of spinal cord compression was characteristic of a focal decrease in ADC and an increase in FA [20,34]. Cheung et al. [35] conducted DTI analysis on CSM rat models and revealed the characteristic increase in ADC and decrease in FA as late as 9 months after the start of compression. In the present study, hardly could we find any correlation between DTI difference and disease duration because seldom studies provided duration of CSM patients.

In this study, a significant decrease in FA was observed not only in the part of most compressed level, but also at sites distant from MCL, like C2-C3 level. This finding reflects the fact that CSM-associated demyelination and axonal damage afflicted both the myelopathic lesion and the distal sites in the chronic course of the disease [36,37]. Thus, the diffusion indexes from the whole cervical spinal cord could be selected to comprehensively reflect overall damage in CSM patients.

Compared with conventional MRI, DTI indexes prove to be more sensitive in the detection of CSM patients, especially early myelopathy. HSI on T2-weighted images is often used to diagnose CSM, but this finding is not observed in every patient with clinical signs of myelopathy, and it sensitivity is reported to be low (between 15% and 65%) [32,38–40]. Additionally, T2 HSI is generally observed only in later stages of the disease [11]. Recent studies conducted DTI analysis on CSM patients with neurological signs but without T2 HSI, and revealed significant reduced FA and increased ADC at the stenotic levels.[41–43] Demir et al. [11] suggested that ADC value had nearly a 80% sensitivity and 53% specificity for detecting myelopathy in patients with spinal cord compression. Uda et al. [25] employed receiver-operator characteristic (ROC) analysis and suggested mean ADC as the best predictor of meyelopahty. Consistent to previous studies, our meta-analysis showed significantly increased ADC and decreased FA at both MCL and normal levels of CSM patients.

In addition to identifying abnormalities before the development of T2 HSI, quantitative analysis of DTI metrics also makes it possible to evaluate severity of myelopathy and predict the outcome of surgical treatments. Previous attempts have been made to investigate the relationship between DTI indexes and various clinical assessments in patients with CSM [14,21,25–27]. Budzik et al. [24] reported a positive correlation with FA and a self-administered questionnaire. Data from Jones et al. [44] demonstrated a strong correlation between FA and specific clinical assessments, including modified Japanese Orthopedic Association (mJOA) and Nurick scores. Recently, they also reported that severely affected CSM patients with higher FA at the compressed level tended to achieve better functional recovery after decompression surgery when compared with subjects with lower FA, indicating FA as a potential biomarker of better postoperative outcome [14]. Similarly, in the study of Wen et al. [21], FA was significantly correlated with mJOA score and enabled prediction of good surgical outcomes by Logistic regression (P = 0.030), while the presence of HSI on T2-weighted images did not (P = 0.176).

There are a number of clinical and methodological confounds that may affect DTI quantitative values. Mamata et al. [12] reported that 46% of the patients of CSM showed no elevation in ADC and no decrease in FA of the spinal cord at the narrow spinal canal level. Meanwhile, they found an increased ADC and a decreased FA with age in the spinal cord. Besides, MRI sequences, magnetic field strengths and ROI placement may also cause differences in the final DTI indexes [27]. DTI of the spinal cord can be performed in any MRI scanner that has a minimum 1.5 T magnet and at least six gradient directions [45]. The more gradient directions, the better the differentiation of nerve fibers. A 3.0 T scanner makes it possible to provide higher quality images because the signal-to-noise ratio correlates with field strength. In addition, movement artifacts are also minimized with a 3.0 T scanner because of the shortened scanning time. Hori et al. [42] investigated the line scan DTI findings in CSM patients using a 0.2-T MRI scanner, and also revealed abnormalities undetectable on T2-weighted images. In the present study, meta-analysis revealed no significant effects of these MRI parameters on the changes of DTI values in CSM patients, while strong effect size variations were found in studies that place ROIs on the sagittal plane.

In addition to improving the diagnosis of CSM and predicting surgical outcome, DTI technology can also be utilized in the controversial management of CSM patients. One possible role for this imaging evaluation would be to assess changes or progression as part of sequential follow-up of patients where the signs and conventional imaging findings are not sufficient to warrant follow-up. It has been well demonstrated that some mild CSM patients can be successfully treated by non-operative treatments [46]. DTI could possibly serve as a non-invasive tool to monitor asymptomatic or mildly affected CSM patients, which does not currently exist.

There are some methodology and data limitations to be considered for this meta-analysis. Firstly, the number of studies and the size of studies were modest, limiting the generalizability of the results. Although meta regression showed that the modest heterogeneity might result from male ratio of the participants, we failed to explore more potential modifiers of DTI changes because of the limited information from the pooled studies. In addition, the stage of CSM disease, either early or advanced, may influence the DTI findings. In the present study, diagnostic criteria of CSM patients we used were clinical manifestations and imaging evidences, which represented a relatively later stage of the disease. An early-stage DTI meta study could be interesting because it would provide stronger evidence that DTI has a better diagnostic ability of early CSM patients without abnormality on conventional MRI.

## Conclusions

In conclusion, our findings from this meta-analysis of DTI studies of CSM patients clearly demonstrated a significant FA reduction and ADC increase compared with healthy subjects. This results support the use of DTI parameters in differentiating CSM patients from health subjects. Future researches are required to investigate the diagnosis performance of DTI in cervical spondylotic myelopathy.

## Supporting Information

S1 PRISMA Checklist PRISMA 2009 for the Meta-analysis. (PDF)
